# Supplementary material for: A fault-intrusion-tolerant system and deadline-aware algorithm for scheduling scientific workflow in the cloud
Source: PeerJ Comput Sci. 2021 Nov 2;7:e747. doi: 10.7717/peerj-cs.747 (PMC8576554; doi:10.7717/peerj-cs.747)
Supplement: Supplemental Information 6 [file peerj-cs-07-747-s006.zip › Myproject1.1/docs/org/cloudbus/cloudsim/core/predicates/package-use.html]

Uses of Package org.cloudbus.cloudsim.core.predicates


JavaScript is disabled on your browser.


- Overview
- Package
- Class
- Use
- Tree
- Deprecated
- Index
- Help

- Prev
- Next

- Frames
- No Frames

- All Classes

# Uses of Package org.cloudbus.cloudsim.core.predicates

- Packages that use org.cloudbus.cloudsim.core.predicates

  | Package | Description |
  | --- | --- |
  |  |  |
  | --- | --- |
  | org.cloudbus.cloudsim.core |  |
  | org.cloudbus.cloudsim.core.predicates |  |
- Classes in org.cloudbus.cloudsim.core.predicates used by org.cloudbus.cloudsim.core

  Class and Description

  Predicate

  Predicates are used to select events from the deferred queue.

  PredicateAny

  A predicate which will match any event on the deferred event queue.

  PredicateNone

  A predicate which will **not** match any event on the deferred event queue.
- Classes in org.cloudbus.cloudsim.core.predicates used by org.cloudbus.cloudsim.core.predicates

  Class and Description

  Predicate

  Predicates are used to select events from the deferred queue.

- Overview
- Package
- Class
- Use
- Tree
- Deprecated
- Index
- Help

- Prev
- Next

- Frames
- No Frames

- All Classes
